# The effect of empirical and laboratory-confirmed tuberculosis on treatment outcomes

**DOI:** 10.1038/s41598-021-94153-0

**Published:** 2021-07-21

**Authors:** Osman Abdullahi, Ngari Moses, Deche Sanga, Willetts Annie

**Affiliations:** 1 Department of Public Health, Pwani University, P.O Box 195, Kilifi, 80108 Kenya; 2 KEMRI/Wellcome Trust Research Programme, P.O Box 230, Kilifi, 80108 Kenya; 3 Kilifi County TB Control Program, P.O Box 9-80108, Kilifi, Kilifi County Kenya

**Keywords:** Epidemiology, Tuberculosis

## Abstract

The World Health Organization (WHO) criteria for diagnosing and treating Tuberculosis (TB) includes clinical signs, therefore not requiring bacteriological laboratory confirmation. In resource-limited settings, including Kenya, this empirical TB treatment is routine practice however limited data exist on patient clinical outcomes when comparing the method of diagnosis. We evaluated TB treatment outcomes comparing clinically diagnosed and bacteriologically confirmed TB, 6 months after starting treatment of TB in a rural county in Kenya. Our analysis compared patients with a clinical versus a bacteriologically confirmed TB diagnosis. In this retrospective analysis, we included all adults (≥ 18 years) starting treatment of TB and followed up for 6 months, within the County TB surveillance database from 2012 to 2018. Patients included from both public and private facilities. The TB treatment outcomes assessed included treatment success, treatment failure, death, defaulted and transferred out. We used survival regression models to assess effect of type of diagnosis on TB treatment outcome defining time at risk from date of starting treatment to experiencing one of the treatment outcomes or completing 6-months of treatment. A total of 12,856 patients; median age 37 [IQR 28 − 50] years were included. 7639 (59%) were male while 11,339 (88%) were pulmonary TB cases. Overall, 11,633 (90%) were given first-line TB treatment and 3791 (29%) were HIV infected. 6472 (50%) of the patients were clinically diagnosed of whom 4521/6472 (70%) had a negative sputum/GeneXpert test. During the study 5565 person-years (PYs) observed, treatment success was 82% and 83% amongst clinically and bacteriologically diagnosed patients (P = 0.05). There were no significant differences in defaulting (P = 0.70) or transfer out (P = 0.19) between clinically and bacteriologically diagnosed patients. Mortality was significantly higher among clinically diagnosed patients: 639 (9.9%) deaths compared to 285 (4.5%) amongst the bacteriologically diagnosed patients; aHR 5.16 (95%CI 2.17 − 12.3) P < 0.001. Our study suggests survival during empirical TB treatment is significantly lower compared to patients with laboratory evidence, irrespective of HIV status and age. To improve TB treatment outcomes amongst clinically diagnosed patients, we recommend systematic screening for comorbidities, prompt diagnosis and management of other infections.

## Introduction

The global targets to reduce deaths attributed to tuberculosis (TB) and new infections remains a primary indicator for a nation to demonstrate progress in fighting the epidemic^1,2^. In 2018 TB was associated with 1.4 million deaths and infected a further 10 million people^1–3^. National TB control programs are required to adopt key strategies that expand access to TB screening and diagnosis^4^. However, inadequate facilities challenge diagnosis within resource-limited countries^5^. In addition, the routine use of unreliable diagnostic methods and empirical treatment within high burden countries directly affects the interpretation of TB treatment outcome data^6^. The real progress towards achieving TB control targets among resource-limited countries is therefore unknown.

For decades, obtaining an accurate TB diagnosis proved challenging^7^ due to reliance on routine methods with variable and imperfect sensitivity, including clinical history, clinical examination, chest radiograph (CXR), sputum smear and culture^8–10^. Recently more sensitive TB diagnostic techniques, including the widely used GeneXpert systems for early diagnosis and treatment has been adopted, however, the increased sensitivity may come at the cost of reduced specificity^11,12^. Furthermore, sensitivity and specificity vary among these methods, especially among children and people living with HIV, potentially leading to misdiagnosis^7,11^. A meta-analysis comparing the accuracy of GeneXpert, Microscopic Observation drug susceptibility assay (MODS) and WHO 2007 algorithm to pulmonary TB among smear-negative patients reported a pooled sensitivity of 67%, 73%, and 61% respectively^13^. Misdiagnosis may indicate TB disease is missed, exposure to the vaccine, those with latent or active disease are not distinguished, or TB may be over-diagnosed – especially when only clinical criteria is applied. A South African study reported 21% of adults dying in hospital with a premortem diagnosis of TB had no TB at autopsy^14^, while in Italy in 1996, 36% of people dying of AIDS and a clinical diagnosis of TB had no evidence of TB at autopsy^15^. Unfortunately, the current global targets do not consider the risks and consequences of misdiagnosis^4^ despite the WHO recommendations of systematic screening programs^16^.

In resource-limited settings, including Kenya, a decision to initiate TB treatment among people without bacteriological laboratory confirmation is dependent on clinical signs and symptoms which are at the discretion of the individual physician^16^. Empiric treatment is also provided when ambulant HIV-positive individuals with two negative sputum smear microscopy tests and chest radiography findings compatible with TB do not respond to broad-spectrum antimicrobial therapy^17^. However, the reported sensitivity and specificity of empirical diagnosis are both highly variable and sub-optimal. In one multicentre trial, sensitivity ranged from 16 to 44.4%, and the specificity ranged from 86.9 to 95.3% across study sites^18^. In a meta-analysis, the pooled sensitivity and specificity of empiric diagnosis was 61% and 69% respectively^13^. Despite this uncertainty, this presumptive practice of TB diagnosis without laboratory evidence complies with WHO treatment recommendations^16^. Empiric treatment may risk unnecessary administration of TB drugs with potential for delays in the diagnosis of conditions other than TB, adverse drug effects, and increasing antimicrobial resistance. However, it is an appropriate strategy in a context where the pre-test probability of TB is high and diagnostic results have inadequate sensitivity or the consequences of withholding TB treatment are serious^6^.

Limited data describe clinical characteristics and TB treatment outcomes between clinically diagnosed and bacteriologically confirmed TB cases despite empiric clinical diagnosis being common in resource-limited countries. Studies on TB outcomes have more commonly compared the performance of GeneXpert versus a sputum smear microscopy test. In our recent analysis of 5-year surveillance data in Kilifi County in Kenya^19^, we found almost half of the patients received empirical TB treatment. In this study, we aimed to systematically evaluate TB treatment outcomes comparing clinically diagnosed and bacteriologically confirmed TB patients, 6 months after starting TB treatment from 2012 to 2018 in Kilifi County, Kenya.

## Methods

### Study setting

We conducted our study in Kilifi County located on the coast of Kenya. Kilifi County has seven sub-Counties namely, Kilifi North, Kilifi South, Ganze, Malindi, Magarini, Rabai, and Kaloleni. From the 2019 census, Kilifi County had an estimated population of 1.4 million (national: 47 million) and approximately 74% reside in rural areas^20^. Subsistence farming is the main economic activity. In 2017, HIV prevalence in Kilifi County was 3.8% (national 4.9%), and 95% of under-fives children had a BCG vaccine in 2020^21,22^. HIV prevalence among TB cases in Kenya (2016) was 16.7%^23^. The Kenyan national incidence of bacteriological confirmed TB was 558 (95% CI 455–662) cases per 100,000 population in 2016; however, this survey did not disaggregate data by county^23^. In the national survey of 2016, only 6/305(2.0%) TB cases were confirmed rifampicin resistance cases^23^.

TB diagnosis and treatment in Kenya and Kilifi County follow the WHO guidelines. In the county, all public and some private health facilities provide TB treatment. At the health facilities sputum samples are collected and sent to the nearest TB diagnostic laboratory for diagnosis. Test results are received back within 48 h. During the study period the 4-module rapid molecular system; GeneXpert MTB/Rif (Cepheid USA), provided TB diagnostic testing at two public hospitals while the rest of the laboratories used smear-microscopy and referrals to GeneXpert sites. All people initiated on TB treatment were systematically tested for HIV. Those testing positive for HIV were counselled and linked with HIV comprehensive care clinics. Data on CD4 counts and adherence to ARVs were not available for this analysis.

Smear microscopy testing was available in all the sub-counties but GeneXpert testing was available only in three sub-counties in the course of the study (Supplementary Table 1). The main Kilifi County referel hospital is located in Kilifi North sub-county. Malindi and Kaloleni sub-counties are served by sub-county level hospitals. The other four sub-counties are served by lower-level hospitals (Supplementary Table 4).

### Study population

Adults ≥ 18 years old starting anti-TB treatment and registered with the Kilifi County, Kenya TB treatment programme. The surveillance includes both inpatient and outpatient TB patients treated within Kilifi county health facilities. After discharge, those started on TB treatment while admitted in hospital are followed up in the community like those started on TB treatment from outpatient clinics. All adults TB patients registered from January 2012 to December 2018 were included in this analysis.

### Study design

Our study retrospectively analysed the TB patients’ clinical and demographic data from a real-time TB treatment surveillance database. The main exposure was clinically diagnosed versus bacteriologically confirmed TB. We also assessed additional exposures, including clinical and demographic features collected at TB treatment initiation. The study outcome was TB treatment outcomes assessed during 6 months after initiating TB treatment: treatment success (cured and treatment completed), treatment failure, death, defaulted and transferred out.

### Data sources/measurement

A TB diagnosis was according to WHO guidelines: a bacteriologically confirmed TB case was defined as any presumptive TB patient with a positive culture, smear microscopy or GeneXpert MTB/RIF. A clinically diagnosed TB case was defined as a presumptive TB patient diagnosed by a clinician or medical practitioner, usually based on abnormal chest radiograph, extrapulmonary cases, suggestive histology and clinical signs like chronic cough, fever, night sweats and weight loss, but not bacteriologically confirmed.

All TB patients in Kilifi County, are registered on an electronic surveillance system (locally defined as TIBU) and started on TB treatment through the County TB treatment programme. Through this surveillance system, systematic data including demographics, clinical findings, type of TB diagnosis and any regular tests were collected since January 2012. Every month people receive treatment by visiting health facilities to collect their drugs. Treatment outcomes are collected until they complete treatment, when the definitive outcome is documented. Patients failing to attend their monthly clinic visits are traced through community health workers. A patient is categorised as ‘defaulted’ if not traced for two consecutive months. Those moving out of Kilifi county are linked with the nearest clinics in their new residence and categorised as ‘transferred out’. Treatment failure was defined as having a positive sputum smear during month 5 of follow-up visit. Cured was defined as having a negative sputum smear test at month 3 and 6 of follow-up. However, sputum smear testing was not systematically conducted during follow-ups, thus these two outcomes (treatment failure and cured) were not evaluated.

### Quantitative variables

No data were missing on our main study variables (type of diagnosis, dates of starting anti-TB treatment or exiting the follow-ups, and treatment outcomes). However, 100 (0.8%), 921 (7.2%) and 140 (1.1%) patients were missing weight, height, and HIV status respectively. For the HIV , an extra category was created; “unknown”, and included in the analysis because the missing status was assumed not to be at random. Missing weight and height were imputed using multiple imputation with the chained equation method. Body Mass Index (BMI) was computed as weight (Kg) divided by square of height (meters) and categorised into three groups following WHO guidelines: undernourished (BMI < 18.5), normal (BMI 18.5 to 25) and overweight (BMI ≥ 25)^24^. Age was categorised into four groups: 18 to 30, 31 to 40, 41 to 50 and 51 + years.

### Statistical methods

Categorical variables, type of TB diagnosis and treatment outcomes were reported as frequencies and percentages. To explore differences between patients diagnosed using clinical signs and bacteriologically, we conducted log-binomial regression analysis with the binary method of diagnosis as the dependent variable and included all features measured at starting TB treatment. To assess the association between type of TB diagnosis and each TB treatment outcome, we performed a single event survival analysis defining time at risk as person-years (PYs) from date of starting anti-TB treatment to date of treatment completion or other treatment outcomes. Since > 90% of the patients were on first-line TB treatment for 6 months and to maintain uniform follow-up time, we truncated time at risk at 6 months for the few who had not completed treatment.

For each treatment outcome, we built the base survival regression model with a type of TB diagnosis (clinical/ bacteriological) as the only exposure in the model. To build multivariable survival regression models, we added apriori confounders to each outcome model. The apriori confounders included in the multivariable models were based on our previous work and systematic reviews^3,19,25^: age, gender, patient type (new/recurrent TB), type of TB (PTB/EPTB), HIV status, treatment regimen, BMI category and year of diagnosis. For the treatment success outcome, all other treatment outcomes (failure, death, default and transferred out) were collapsed into one group creating a binary outcome. The collapsed level was considered to be informatively censored and thus treated as competing event with treatment success, therefore to assess whether the type of TB diagnosis was associated with treatment success, we used the Fine & Gray competing risk model and reported sub-distribution hazard ratios^26^. For the other outcomes (death, default and transferred out), we assumed non-informative censoring and right censored each patient at the date of respective event or after 6 months. Each outcome was analysed separately including other patients with the other outcomes up to their event date. We assessed proportional hazard assumption (PH) using the Schoenfeld residuals before performing Cox Proportional regression analysis. Where there was evidence of violation of PH assumption, we assessed the survival parametric models that fitted the distribution of our data using the Akaike information criterion (AIC). Probability distributions assessed for the survival analysis parametric models included: Exponential, Weibull, Log-logistic, Gompertz and Log-normal distributions. The parametric distribution with the minimum AIC was considered as the best fitting to our data and selected. The PH assumption was only violated for the treatment default outcome (P = 0.004). After assessing the parametric models, Gompertz distribution had the minimum AIC and was used to analyse this outcome (Supplementary materials: Supplementary Table 2 and 3). We tested the effect modification of HIV status on the association between type of TB diagnosis and each treatment outcome by comparing regression models with and without interaction terms using the likelihood ratio test. We also assessed effect modification of age on the association between type of TB diagnosis and each treatment outcome using the likelihood ratio test. Where we found evidence of effect modification, interaction terms were included in the multivariable models and explored separately in stratified analysis. In sub-analysis, we stratified the above analysis by type of TB (either pulmonary (PTB) or extra pulmonary TB (EPTB)) because extra pulmonary TB were more likely to be diagnosed clinically. All the regression models included the sub-counties within Kilifi County as random effect component to account for the clustering and unobserved heterogeneity^27^. All the statistical analyses were performed using STATA/IC version 15.1 (StataCorp, College Station, TX, USA).

### Study size

With 12,856 patients, assuming mortality of 5.5%^19^ and a two-sided alpha level of 0.05, our study had power > 90%, to detect hazard ratio of ≥ 2.0 of death between patients diagnosed with clinical signs and bacteriologically with ≥ 708 expected deaths in the study.

### Ethical considerations

We obtained ethical approval from Pwani University Ethical Review Board and permission granted by the Kilifi County Ethical Research Committee to access the anonymised patient TB surveillance data. All study participants provided written consent. Design and reporting of the study followed the Strengthening Reporting of Observational Studies in Epidemiology (STROBE) guidelines for reporting observational studies.

## Results

### Patient characteristics

We included 12,856 patients starting TB treatment. Their median (IQR) age was 37 (28–50) years, and 7639 (59%) were male. Most patients were new cases, 11,469 (89%), recruited from a public health facility 10,129 (79%) and pulmonary TB cases 11,339 (88%). A total of 11,315 (88%) patients were on family-based direct observation treatment. Approximately half of the patients had a normal BMI. Overall, 11,633 (90%) were treated with rifampin, isoniazid, pyrazinamide and ethambutol for the 2 months intensive phase, followed by rifampin and isoniazid for the subsequent 4 months (2RHZE/4RH). Three thousand seven hundred and ninety-one (29%) patients had a comorbidity of HIV infection, of which 3594/3791 (95%) were on ARV treatment, and 3764/3791 (99%) were on co-trimoxazole prophylaxis Table 1. HIV infections were significantly different across the sub-counties, it was lowest in Rabai (18%) and highest in Kaloleni (34%) P < 0.001. Background information across the sub-counties are shown in Supplementary Table 4.

**Table 1:** Study participants characteristics at the time of starting anti-TB treatment.

Features	Clinical signs diagnosis (N = 6472)	Bacteriological diagnosis (N = 6384)	All patients (N = 12,856)
**Age in years**			
18 to 30 years	1772 (27)	2460 (39)	4232 (33)
31 to 40 years	1676 (26)	1858 (29)	3534 (27)
41 to 50 years	1153 (18)	1001 (16)	2154 (18)
51 + years	1871 (29)	1065 (17)	2936 (23)
**Sex**			
Male	3450 (53)	4189 (66)	7639 (59)
Female	3022 (47)	2195 (34)	5217 (41)
**Patient type**			
New cases	5770 (89)	5699 (89)	11,469 (89)
Re-treatment cases	702 (11)	685 (11)	1387 (11)
**TB type**			
Pulmonary	5009 (77)	6330 (99)	11,339 (88)
Extrapulmonary	1463 (23)	54 (0.9)	1517 (12)
**Recruitment health facility**			
Public	5063 (78)	5066 (79)	10,129 (79)
Private	1273 (20)	1221 (19)	2494 (19)
Prisons	136 (2.1)	97 (1.5)	233 (1.8)
**DOT**			
Family-based	5,849 (90)	5,466 (86)	11,315 (88)
Community volunteer	268 (4.1)	545 (8.5)	813 (6.3)
Health worker	355 (5.5)	373 (5.8)	728 (5.7)
**Nutrition status**			
Undernourished	1761 (27)	2256 (35)	4017(31)
Normal BMI	3674 (57)	3399 (53)	7073 (55)
Overweight	1037 (16)	729 (11)	1766 (14)
**HIV status**			
HIV uninfected	4161 (64)	4764 (75)	8925 (69)
HIV infected on ARVS	2113 (33)	1481 (23)	3594 (28)
HIV infected not on ARVS	115 (1.8)	82 (1.3)	197 (1.5)
Unknown HIV status	83 (1.3)	57 (0.9)	140 (1.1)
**Treatment regimen**			
2RHZE/4RH	5811 (90)	5824 (91)	11,635 (91)
2SRHZE/1RHZE/5RHE	562 (8.7)	506 (7.9)	1068 (8.3)
2RHZ/4RH	48 (0.7)	52 (0.8)	100 (0.8)
Others	51 (0.8)	2 (0.03)	53 (0.4)
**Sub county**			
Kilifi North	1206 (19)	1157 (18)	2363 (18)
Kilifi South	689 (11)	1425 (22)	2114 (16)
Kaloleni	1437 (22)	1028 (16)	2465 (19)
Malindi	1619 (25)	1493 (23)	3112 (24)
Magarini	843 (13)	652 (10)	1495 (12)
Ganze	354 (5.5)	302 (4.7)	656 (12)
Rabai	324 (5.0)	327 (5.1)	651 (5.1)
**Year of diagnosis**			
2012	1156 (18)	827 (13)	1983 (15)
2013	1040 (16)	848 (13)	1888 (15)
2014	1185 (18)	868 (14)	2053 (16)
2015	704 (11)	981 (15)	1685 (13)
2016	604 (9.3)	898 (14)	1502 (12)
2017	675 (10)	964 (15)	1639 (13)
2018	1108 (17)	998 (16)	2106 (16)

*DOT* direct observed treatment, *BMI* body mass index, *ARVs* antiretroviral.

### Diagnostic method

Of the 12,856 TB patients, 6,472 were clinically diagnosed: 50% (95% CI 49–51%). Of these, 1724/6472 (27%) were diagnosed by abnormal chest X-ray suggestive of TB and 4748/6472 (73%) by WHO clinical symptoms (individual signs not available) (Table 2). A total of 4521/6472 (70%) included negative sputum or GeneXpert MTB/RIF results.

**Table 2:** Diagnosis of TB at the time of starting anti-TB treatment.

TB diagnosis	All patients (N = 12,856)
**Type of diagnosis, N (%)**	
Clinically diagnosed TB	6472 (50)
Bacteriologically confirmed TB	6384 (50)
**Specific signs/test for individual diagnosis**	
Clinically diagnosed TB (N = 6472)	
Abnormal chest X-ray indicative of TB	1724 (27)
WHO clinical symptoms^a^	4748 (73)
Negative sputum or GeneXpert^b^	4521 (70)
Bacteriological confirmed TB (N = 6384)	
Sputum smear microscopy-positive	4119 (65)
GeneXpert MTB/RIF for sputum positive	1258 (20)
Both sputum and GeneXpert positive	1007 (16)

^a^Individual WHO clinical signs not available.

^b^Proportion of the total clinically diagnosed TB cases who had a negative sputum or GeneXpert test.

There were 6384 bacteriologically confirmed TB cases: 50% (95% CI 49–51%). A total of 4119/6384 (64%) patients, had a positive sputum smear microscopy, 1258/6384 (20%) had a positive GeneXpert MTB/RIF for sputum while 1700/6384 (16%) had both positive sputum and GeneXpert MTB/RIF. The participants’ characteristics at the time of starting TB treatment were stratified by TB diagnosis, as shown in Table 1 above.

Clinical diagnosis of TB was positively assiocated with age, EPTB (aRR 2.09 (95%CI 1.71–2.56)), a prison health facility and HIV infection (aRR 1.21 (95%CI 1.14–1.28)) Supplementary Table 5.

### TB treatment outcome

The 12,856 patients were in follow-up for 5,565 Person-Years (PYs). Overall, 10,601/12,856 (82%) patients successfully completed TB treatment: 82% and 83% among clinically and bacteriologically diagnosed TB patients, respectively. Of the 10,601 successfully treated patients, 2451 (23%) were ‘cured’ and 8150 (77%) completed treatment (Table 3).

**Table 3:** TB treatment outcomes after 6 months of anti-TB treatment.

TB treatment outcome	Clinical signs diagnosis (N = 6472)	Bacteriological diagnosis (N = 6384)	All patients (N = 12,856)
Treatment success	5276 (82)	5325 (83)	10,601 (82)
Cured^a^	_–_	2451 (46)	2451 (23)
Treatment completed^b^	5276 (100)	2874 (54)	8150 (77)
Treatment failure	5 (0.08)	93 (1.5)	98 (0.8)
Died	639 (9.9)	285 (4.5)	924 (7.2)
Defaulted/lost-to-follow-up	389 (6.0)	471 (7.4)	860 (6.7)
Transfer out	163 (2.5)	210 (3.3)	373 (2.9)

^a^Proportion of treatment success defined following WHO guideline.

^b^Proportion of treatment success defined following WHO guideline.

**Figure 1:**
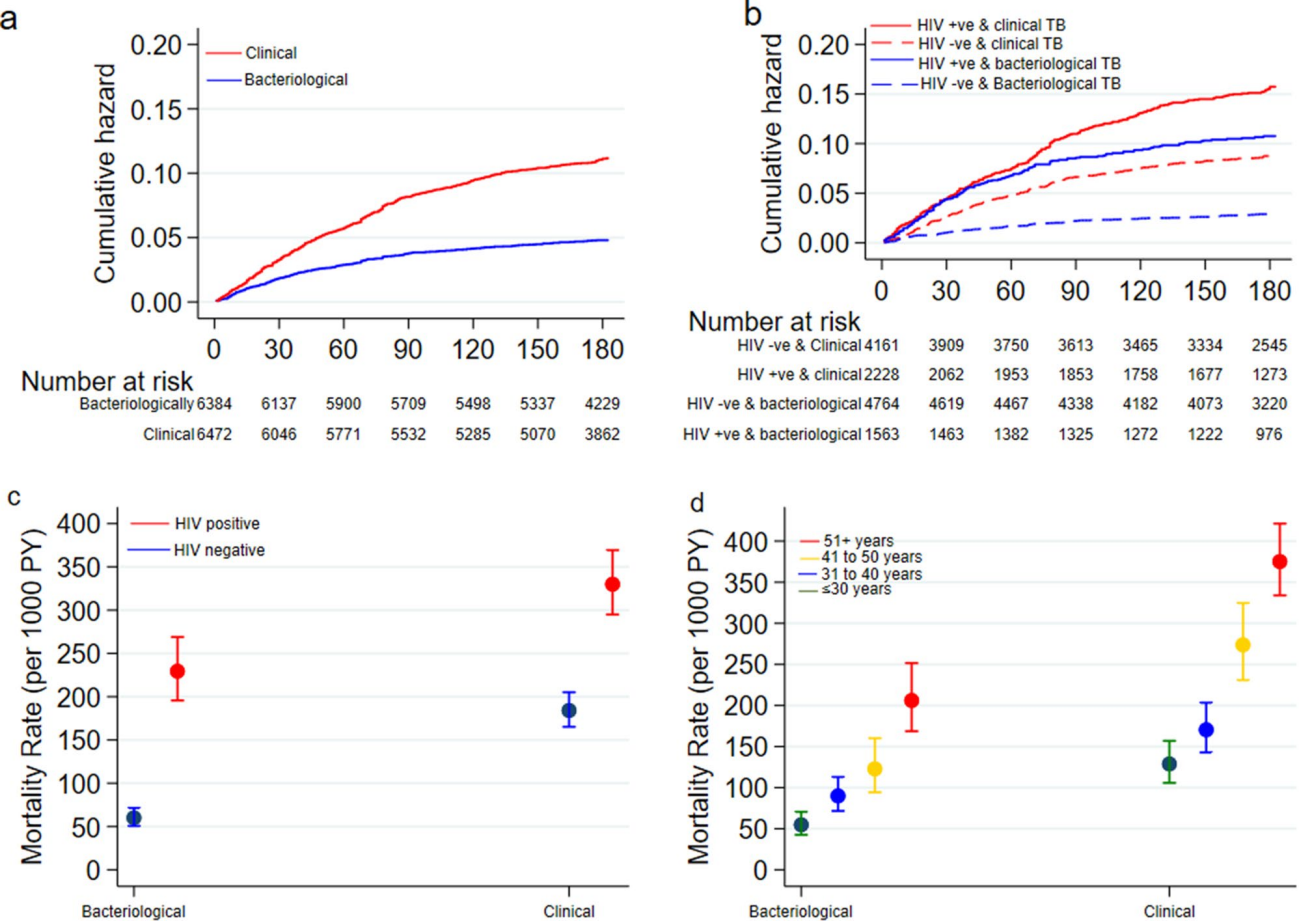
**(A)** Cumulative hazard of deaths stratified by type of TB diagnosis;** (B) **cumulative hazard of deaths stratified by type of TB diagnosis with HIV status;** (C) **mortality rate stratified by type of TB diagnosis with HIV status and** (D) **mortality rate stratified by type of TB diagnosis with age groups.

Overall, 924 (7.2%) patients died: 639 (9.9%) among clinically diagnosed patients and 285 (4.5%) among bacteriologically confirmed TB cases. The overall mortality rate was 166 (95% CI 156 to 177) deaths per 1000 PYs: 234 (95% CI 216 to 253) and 101 (95% CI 90 to 113) deaths per 1000 PYs among clinically and bacteriologically diagnosed TB patients, respectively. The median time to death was 47 days: 51 days versus 40 days amongst the clinically and bacteriologically diagnosed TB patients respectively (log-rank test P-value < 0.001) Fig. 1A. However, among the EPTB, the median days to death was 49 days versus 62 days amongst the clinically and bacteriologically diagnosed TB patients. The risk of death was higher among the EPTB compared to PTB patients (aHR 2.21 (95%CI 1.91–2.54)). The proportions of clinical versus bacteriological diagnosis and deaths varied by sub-county (p < 0.001): the most urban sub-county (Kilifi South) had 33% empirical diagnosis and 6% deaths compared with 58% empiric diagnosis and 13% deaths at rural Kaloleni sub-county (Supplementary Table 6).

Eight hundred and sixty (6.7%) patients defaulted treatment: 389 (6.0%) among clinically diagnosed patients and 471 (7.4%) among bacteriologically confirmed TB cases.

Three hundred and seventy-three (2.9%) patients were transferred out; 163 (2.5%) among clinically diagnosed patients and 210 (3.3%) among bacteriologically confirmed TB cases (Table 3).

### Association between TB diagnosis type and treatment outcome

Clinically diagnosed TB had a borderline effect on the binary outcome of TB treatment success: crude sub-distribution Hazard ratio (SHR) 1.07 (95% CI 0.99 to 1.14) and adjusted SHR 1.06 (95% CI 1.01 to 1.11) compared to bacteriologically confirmed TB cases.

However, clinically diagnosed TB was associated with mortality: crude hazards ratio (CHR) 2.18 (95% CI 1.89 to 2.51). We found evidence that the HIV status (P < 0.001) and age (P = 0.006) modified the effect of type of TB diagnosis on mortality and included their interaction terms in the multivariable model (Supplementary Table 2). Clinically diagnosed TB was associated with mortality in the multivariable model; adjusted hazards ratio (aHR) 5.16 (95% CI 2.17 to 12.3) compared to bacteriologically confirmed TB cases (Fig. 1A and Table 4).

**Table 4:** Univariate and multivariate analysis of TB treatment outcomes associated with diagnosis of TB at time of starting anti-TB treatment.

TB treatment outcome	Univariate analysis (base models)		Multivariate analysis	
	Crude SHR (95% CI)	P-value	Adjusted SHR (95% CI)^a^	P-value
Treatment success	1.07 (0.99 to 1.14)	0.07	1.06 (1.01 to 1.11)^d^	0.05

	Crude HR (95% CI)		Adjusted HR (95% CI)^a^	
Treatment failure^c^				
Died	2.18 (1.89 to 2.51)	< 0.001	5.16 (2.17 to 12.3)^e^	< 0.001
Defaulted/lost-to-follow-up^b^	0.96 (0.84 to 1.11)	0.61	0.97 (0.83 to 1.13)	0.70
Transfer out	0.84 (0.68 to 1.04)	0.10	0.85 (0.68 to 1.08)	0.19

^a^Adjusted for apriori confounders: age, gender, patient type, TB type (P/EP), HIV status, treatment regimen and BMI groups, *SHR* sub-distribution hazard ratios from Fine & Gray competing risk regression model, *HR* hazard ratios from Cox proportion regression models.

^b^Hazard ratios are from the Gompertz parametric regression model.

^c^No measures of association was estimated for treatment failure because of obvious bias in classifying the clinically diagnosed patients who had a negative sputum test when starting treatment.

^d^The adjusted regression models included the HIV interaction term.

^e^The adjusted regression models included the HIV, age interaction terms.

Clinically diagnosed TB was not associated with treatment default: aHR 0.97 (95% CI 0.83 to 1.13) or transfer out: aHR 0.85 (95% CI 0.68 to 1.08) compared to bacteriologically confirmed TB cases (Table 4).

In the sub-analysis including only the clinically diagnosed TB cases, treatment success was significantly higher (P < 0.001), but mortality (P = 0.03) and default (P < 0.001) were lower among patients with a negative sputum microscopy or GeneXpert test compared to those with no test result (Supplementary Table 7).

In the sub-analysis of patients with known HIV status, TB treatment success was significantly higher among HIV negative patients: 84% among clinically diagnosed TB and 85% among bacteriologically confirmed TB cases compared to the HIV infected: 78% among both clinically and bacteriologically diagnosed TB patients (P < 0.001). The mortality rate was higher among HIV infected patients. However, clinically diagnosed TB was associated with higher rate ratio among both the HIV infected (aRR 1.35 (95 CI 1.10 to 1.65) and not infected (aRR 2.15 (95% CI 1.74 to 2.67) compared to bacteriologically confirmed TB cases (Fig. 1B,C and Supplementary Table 8). The mortality rate was highest among the older patients (51 + years) and significantly higher among clinically diagnosed TB compared to bacteriologically confirmed TB cases in all the age groups in the stratified analysis (Fig. 1D and Supplementary Table 8).

Tretament outcomes stratified by type of TB (either PTB or EPTB) are shown in Supplementary Table 9. Among the PTB patients, clinically diagnosed TB was associated with mortality; aHR 7.06 (95% CI 2.79 to 12.9) compared to bacteriologically confirmed TB cases but not among the EPTB patients:aHR 0.16 (95% CI 0.01 to 3.09) Supplementary Table 10. All other treatment outcomes were not significantly different among PTB and EPTB patients.

## Discussion

In this large study of TB patients systematically followed-up for 6 months while on treatment, we observed a significantly higher rate of mortality among people receiving empirical TB treatment compared to bacteriologically confirmed cases. Overall, while the treatment success rates were similar between clinically diagnosed and bacteriologically confirmed TB cases, the mortality rate was fivefold higher (p < 0.001) among clinically diagnosed patients in a multivariable analysis that adjusted for age, gender, new/retreatment case, TB type, HIV status, treatment regimen and BMI. However there was no significant difference in other treatment outcomes between the two diagnostic types.

We found approximately 50% of patients were diagnosed clinically and treated empirically without laboratory confirmation, of which 70% had negative sputum smear or GeneXpert MTB/RIF results. Our empirical diagnostic rate is approximately similar to the WHO level of 43% of all cases reported to WHO globally in 2017^28^. Previous studies suggest high proportions of empiric diagnosis is likely to lead to an increase in false-positive TB. For example, in a simulated study, the use of microscopy and clinical diagnosis could result in 37% false positivity during 2017–2020^29^. Studies suggests most false-positive TB cases potentially have other diseases. In a Malawian study with a high HIV seroprevalence rate of 89%, 61% of TB patients were either possible TB cases or had other non-TB diagnoses^30^. After follow up of patients for eight months, 53% died among the patients with non-TB diagnoses versus 31% amongst the bacteriologically confirmed TB cases^30^. In Uganda however, the proportion of TB patients with clinically diagnosed pulmonary TB was 21%^31^. Reasons for the high frequency of empirical treatment practices are not explored in our study however, 70% of this group received bacteriological testing. This finding suggests the tests either have a low specificity in this context, or overdiagnosis due to a trust in clinical signs rather than the negative bacteriological result. The latter suggested in one study in India in which TB diagnosis was more reliant on clinical opinion and less on bacterial confirmation^32^.

We had expected the high risk of death among clinically diagnosed TB patients in this study to be entirely driven by HIV infection, however, after stratification by the HIV status, the higher risk of death among the clinically diagnosed patients remained. Similarly, the risk of death was consistently higher among clinically diagnosed TB across all the age groups and among pulmonary TB cases compared to etra-pulmonary TB cases. However, further analysis of the clinically diagnosed TB shows those with a negative sputum microscopy or GeneXpert MTB/RIF results had a higher treatment success rate and marginally lower mortality compared to those not tested. The finding of variation in proportions of study participants with clinical versus bacteriological in the various sub-counties and their case fatality potentially explained by variation in the availability of diagnostic facilities, the experience of clinical and laboratory staff in TB diagnosis and access to the health facility^33^. Similarly, the explanation for our observation of bacteriologically diagnosed having higher time to death compared to clinically diagnosed (median of 62 days vs. 49 days respectively) among EPTB patients is unclear and we could not interrogate the data further due to limited number of bacteriologically diagnosed cases (only 3.5%).

National programmes define deaths among people receiving TB treatment as ‘tuberculosis disease’. While it is reasonable to assume TB is the causative agent among people with clear signs and symptoms of pulmonary disease with a sputum smear or GeneXpert positive result, the empirical treatment leaves large margins of doubt. This is especially in HIV-infected patients whose diagnosis is complicated by decreased likelihood of sputum smear positivity and increase in respiratory diseases^7^. These include diseases related to HIV (e.g. *Pneumocystis carinii* pneumonia, fungal infection or bronchiectasis) and diseases unrelated to HIV (e.g. heart failure)^34–37^. In Uganda, the sensitivity and specificity of empirical diagnosis among HIV-infected smear-negative TB patients was 63% and 74% respectively, and only 35.3% of empirically treated patients had microbiologically confirmed TB^38^. Similarly, in Tanzania only 28% of empirically treated patients had microbiologically confirmed TB^39^. In resource-limited settings with a high burden of TB/HIV coinfection, as found in Kenya, the decision to treat smear-negative patients empirically is mainly driven by the emphasis in management guidelines to avoid consequences of false-negative TB diagnosis and treatment delays^30,40^.

It is plausible that the higher mortality observed is due to the inclusion of patients who have severe illnesses, other than TB, for which they received inappropriate or inadequate treatment^41^. This view was supported by two national samples of TB patients in Kenya in the 1960s and 1970s. Mortality was lower among individuals with positive cultures than among those with negative cultures, or who failed to produce sputum specimens^42^. Other TB programmes have provided evidence of patients clinically diagnosed with TB but confirmed not to have TB at autopsy both among HIV infected and non-infected patients^43^. Studies in South Africa have found high prevalence of non-communicable diseases (NCD) among TB patients on treatment^44^. Similar studies using the chest radiograph, a popular tool to clinically diagnose TB, report a high sensitivity (96%) however a very low specificity (46%)^45^. In Africa and Asia, misdiagnosis and treatment of TB is a significant threat in delaying diagnosis and starting treatment for cancer (notably lymphoma and lung cancer)^46–51^. Our findings indicate that many of the clinically diagnosed TB might be suffering from other severe infections with overlapping clinical signs of TB^14,34,41,52–54^. The magnitude of other diseases among people with a TB diagnosis is, however, unknown. The above studies may suggest that mortality is strongly associated with diagnostic uncertainty.

The real effect of overdiagnosis of TB includes withholding appropriate management for the actual condition, and undergoing prolonged anti-tuberculosis therapy with its known potential adverse effects, and early death. Social consequences of a person and their family receiving a TB diagnosis include community stigma and psychological costs of anxiety amongst people who believe themselves exposed to infection^55^. Financial costs of overdiagnosis, associated with unnecessary contact tracing and investigations and inappropriate prophylaxis, further limit overwhelmed health systems in resource-poor settings^56^. Future research is recommended to improve our understanding of the underlying causes of the high mortality amongst the clinically diagnosed TB patients by systematically screening for other diseases in sub-Saharan Africa settings.

The main strength of our study is the large size with adequate events and systematic follow-up for the 6 months. The study limitations include data only from one county within Kenya therefore, we are unable to generalise to other counties in Kenya or Africa. This passive surveillance study design and lack of systematic collection of data on routine tests, including CD4 counts for HIV infected and other potential comorbidity to inform our analyses. The data available to us did not have a variable to differentiate those starting TB treatment during hospital admission or from outpatient clinics and therefore we could not assess if mortality rates were different between the two groups. Finally, there was no systematic data on causes of deaths, adherence to the TB treatment and testing of anti-TB drug resistance.

## Conclusion

Our study suggests that survival during empirical TB treatment is significantly lower compared to patients with laboratory evidence, irrespective of HIV status and age. Other TB treatment outcomes are not different. To promptly diagnose for other infections and avoid treatment delay, we recommend systematic screening and treatment for comorbidities among the clinically diagnosed TB patients.

## Supplementary Information

Supplementary Tables.

## Data Availability

The datasets used in the study are available from the corresponding author on reasonable request.

## Abbreviations

aHR: Adjusted hazards ratio
AIC: Akaike information criterion
AIDS: Acquired immunodeficiency syndrome
ARVs: Antiretroviral
BCG: Bacillus Calmette–Guérin
BMI: Body mass index
CD4: Cluster of differentiation 4
CHR: Crude hazards ratio
CXR: Chest radiograph
EPTB: Extra pulmonary tuberculosis
HIV: Human immunodeficiency virus
IQR: Interquartile range
MODS: Microscopic observation drug susceptibility
MTB: Mycobacterium tuberculosis
NCD: Non-communicable diseases
PH: Proportional hazard
PTB: Pulmonary tuberculosis
PYs: Person-years
SHR: Sub-distribution hazard ratio
TB: Tuberculosis
WHO: World Health Organization
