# Supplementary material for: The effect of empirical and laboratory-confirmed tuberculosis on treatment outcomes
Source: Sci Rep. 2021 Jul 21;11:14854. doi: 10.1038/s41598-021-94153-0 (PMC8295390; doi:10.1038/s41598-021-94153-0)
Supplement: Supplementary file 1 — Supplementary Tables. [file 41598_2021_94153_MOESM1_ESM.docx]

**The effect of empirical and laboratory-confirmed Tuberculosis on treatment outcomes.**

Osman Abdullahi, Ngari Moses, Deche Sanga, Willetts Annie

**Supplementary materials**

Supplementary Table 1. **Number of health facility with capacity to test TB using microscopy or GeneXpert in the sub-counties.**

|  | Number of health facilities | | | | | | |
| --- | --- | --- | --- | --- | --- | --- | --- |
|  | 2012 | 2013 | 2014 | 2015 | 2016 | 2017 | 2018 |
| Microscopy | | | | | | | |
| Kilifi North | 3 | 3 | 5 | 5 | 7 | 7 | 7 |
| Kilifi South | 3 | 3 | 4 | 5 | 6 | 6 | 7 |
| Kaloleni | 3 | 3 | 4 | 6 | 6 | 6 | 5 |
| Malindi | 5 | 5 | 7 | 7 | 8 | 8 | 8 |
| Magarini | 3 | 3 | 5 | 6 | 6 | 7 | 7 |
| Ganze | 3 | 3 | 3 | 3 | 3 | 4 | 4 |
| Rabai | 2 | 2 | 2 | 2 | 2 | 2 | 2 |
| GeneXpert | | | | | | | |
| Kilifi North | 0 | 0 | 1 | 1 | 1 | 1 | 1 |
| Kaloleni | 0 | 0 | 0 | 0 | 0 | 1 | 1 |
| Malindi | 1 | 1 | 1 | 1 | 1 | 1 | 1 |

Supplementary Table 2. **Test of Proportion hazard assumption and interaction.**

| **TB treatment outcome** | **PH test P-value** | **Test of HIV interaction P-value** | **Test of age interaction P-value** |
| --- | --- | --- | --- |
| Treatment success | 0.55 | 0.0005 | 0.75 |
| Treatment failure |  |  |  |
| Died | 0.11 | <0.001 | 0.006 |
| Default | 0.004 | 0.37 | 0.19 |
| Transfer out | 0.40 | 0.74 | 0.94 |
| *Fine & Gray competing risk model used, Proportion hazard (PH) assumption P-value from the Schoenfeld residuals test, test of HIV, age and type of TB diagnosis interaction p-value form likelihood ratio test. | | | |

Supplementary Table 3. **Section of survival analysis parametric model using Akaike information criterion (AIC).**

| **Parametric distributions** | **AIC values** | **Parametric distributions selected** |
| --- | --- | --- |
| Exponential | 8120.44 | No |
| Weibull | 8108.92 | No |
| Log-logistic | 8104.99 | No |
| Gompertz | 8070.30 | Selected (minimum AIC) |
| Log-normal | 8086.47 | No |
| AIC-Akaike information criterion, Gompertz distribution selected for the TB treatment outcome that violated the proportion hazard assumption (treatment default). | | |

Supplementary Table 4. **Selected features and treatment outcomes across the sub-counties.**

| **Sub-county** | **Number of patients** | **Number of health facilities** | **Type of main health facility** | **% HIV infected** | **% Clinical diagnosis** | **% treatment success** | **% Deaths** |
| --- | --- | --- | --- | --- | --- | --- | --- |
| Kilifi North | 2,363 (18) | 29 | Host the Kilifi County referral hospital. | 738 (31) | 1206 (51) | 1907 (81) | 193 (8.2) |
| Kilifi-South* | 2,114 (16) | 16 | Served by lower-level hospitals | 650 (31) | 689 (33) | 1756 (83) | 81 (3.8) |
| Kaloleni | 2,465 (19) | 17 | Host a sub-country hospital | 849 (34) | 1437 (58) | 1998 (81) | 251 (10) |
| Malindi | 3,112 (24) | 21 | Host a sub-country hospital | 939 (30) | 1619 (52) | 2542 (82) | 174 (5.6) |
| Magarini | 1,495 (12) | 27 | Served by lower-level hospitals | 355 (24) | 843 (56) | 1290 (86) | 99 (6.6) |
| Ganze | 656 (12) | 16 | Served by lower-level hospitals | 142 (22) | 354 (54) | 559 (85) | 57 (8.7) |
| Rabai | 651 (5.1) | 12 | Served by lower-level hospitals | 118 (18) | 324 (50) | 549 (84) | 69 (11) |
| Total | 12,856 |  |  | 3791 (29) | 6472 (50) | 10601 (82) | 924 (7.2) |
| *Home to Mtwapa, a centre of commercial sex workers. | | | | | | | |

Supplementary Table 5: **Factors associated with clinical diagnosis of TB.**

| **Features** | **Univariate analysis** | | **Multivariable analysis** | |
| --- | --- | --- | --- | --- |
|  | **Crude RR (95% CI)** | **P-value** | **Adjusted RR (95% CI)** | **P-value** |
| Age in years | 1.15 (1.10–1.20) | <0.001 | 1.15 (1.11–1.18) | <0.001 |
| Sex |  |  |  |  |
| Male | Reference |  | Reference |  |
| Female | 1.28 (1.20–1.37) | <0.001 | 1.22 (1.15–1.30) | <0.001 |
| Patient type |  |  |  |  |
| New cases | Reference |  | ¶ |  |
| Re-treatment cases | 1.01 (0.88–1.15) | 0.93 | ¶ |  |
| TB type |  |  |  |  |
| Pulmonary | Reference |  | Reference |  |
| Extrapulmonary | 2.18 (1.75–2.72) | <0.001 | 2.09 (1.71–2.56) | <0.001 |
| Recruitment health facility |  |  |  |  |
| Public | Reference |  | Reference |  |
| Private | 1.02 (0.86–1.21) | 0.81 | 1.01 (0.91–1.12) | 0.89 |
| Prisons | 1.17 (1.01–1.35) | 0.03 | 1.45 (1.28–1.65) | <0.001 |
| DOT |  |  |  |  |
| Family-based | Reference |  | Reference |  |
| Community volunteer | 0.64 (0.48–0.84) | 0.002 | 0.67 (0.56–0.81) | <0.001 |
| Health worker | 0.94 (0.78–1.15) | 0.56 | 0.91 (0.81–1.03) | 0.14 |
| Nutrition status |  |  |  |  |
| Undernourished | 0.84 (0.80–0.89) | <0.001 | 0.93 (0.89–0.97) | <0.001 |
| Normal BMI | Reference |  | Reference |  |
| Overweight | 1.13 (1.02–1.26) | 0.02 | 0.99 (0.90–1.10) | 0.87 |
| HIV status |  |  |  |  |
| HIV uninfected | Reference |  | Reference |  |
| HIV infected on ARVS | 1.26 (1.18–1.34) | <0.001 | 1.21 (1.14–1.28) | <0.001 |
| HIV infected not on ARVS | 1.25 (1.11–1.41) | <0.001 | 1.09 (0.97–1.23) | 0.15 |
| Unknown HIV status | 1.27 (1.08–1.50) | 0.004 | 1.11 (0.93–1.31) | 0.26 |
| Treatment regimen |  |  |  |  |
| 2RHZE/4RH | Reference |  | ¶ |  |
| 2SRHZE/1RHZE/5RHE | 1.05 (0.87–1.27) | 0.59 | ¶ |  |
| 2RHZ/4RH | 0.96 (0.80–1.16) | 0.67 | ¶ |  |
| Others | 1.93 (1.65–2.25) | <0.001 | ¶ |  |
| Year of diagnosis |  |  |  |  |
| 2012 | Reference |  | Reference |  |
| 2013 | 0.94 (0.82–1.09) | 0.45 | 0.93 (0.83–1.05) | 0.24 |
| 2014 | 0.99 (0.94–1.04) | 0.69 | 0.96 (0.91–1.01) | 0.09 |
| 2015 | 0.72 (0.55–0.93) | 0.01 | 0.73 (0.58–0.91) | 0.006 |
| 2016 | 0.69 (0.59–0.81) | <0.001 | 0.67 (0.57–0.78) | <0.001 |
| 2017 | 0.71 (0.65–0.77) | <0.001 | 0.70 (0.63–0.78) | <0.001 |
| 2018 | 0.90 (0.83–0.98) | 0.01 | 0.86 (0.79–0.95) | 0.003 |
| Multivariable model performance |  |  |  |  |
| AUC (95% CI) |  |  | 0.74 (0.73–0.75) | |
| DOT-Direct observed treatment, BMI-Body Mass Index, ARVs-Antiretroviral, ¶; Variables not selected for inclusion in the multivariable model using backward stepwise method retaining only those with a P<0.1, RR; relative risk, AUC; Area Under the ROC Curve, RR are from the log-binomial regression models. | | | | |

Supplementary Table 6. **Proportions of study participants from each sub-county and the deaths.**

| **Sub-county** | **Clinical signs diagnosis (N=6,472)** | | **Bacteriological diagnosis (N=6,384)** | | **Total (N=12856)** |
| --- | --- | --- | --- | --- | --- |
|  | N (%) | Deaths (%) | N (%) | Deaths (%) |  |
| Kilifi North | 1206 (51) | 142 (12) | 1157 (49) | 51 (4.4) | 2363 |
| Kilifi South | 689 (33) | 42 (6.1) | 1425 (67) | 39 (2.7) | 2114 |
| Kaloleni | 1437 (58) | 181 (13) | 1028 (42) | 70 (6.8) | 2465 |
| Malindi | 1619 (52) | 110 (6.8) | 1493 (48) | 64 (4.3) | 3112 |
| Magarini | 843 (56) | 71 (8.4) | 652 (44) | 28 (4.3) | 1495 |
| Ganze | 354 (54) | 43 (12) | 302 (46) | 14 (4.6) | 656 |
| Rabai | 324 (50) | 50 (15) | 327 (50) | 19 (5.8) | 651 |

Supplementary Table 7. **TB treatment outcomes among the clinically diagnosed TB cases.**

| **TB treatment outcome** | **Clinically diagnosed TB (N=6,472)** | | |
| --- | --- | --- | --- |
|  | **No sputum or GeneXpert test (N=1,951)** | **Negative sputum or GeneXpert (N=4,521)** | **P-value*** |
| Treatment success | 1,517 (78) | 3,759 (83) | <0.001 |
| Treatment failure | 1 (0.05) | 4 (0.09) | 0.62 |
| Died | 217 (11) | 422 (9.3) | 0.03 |
| Default | 158 (8.1) | 231 (5.1) | <0.001 |
| Transfer out | 58 (3.0) | 105 (2.3) | 0.13 |
| *P-value form chi-square or Fisher’s exact test. | | | |

Supplementary Table 8. **Mortality rates stratified by HIV status and age.**

| **Category** | **N** | **Deaths**  **N (%)** | **Mortality rate**  **(95% CI)/1000 PY** | **Mortality rate ratios** |
| --- | --- | --- | --- | --- |
| **HIV status*** | | | | |
| HIV Negative |  |  |  |  |
| Clinical diagnosis | 4,161 | 328 (7.9) | 184 (165 to 205) | 2.15 (1.74 to 2.67) |
| Bacteriological diagnosis | 4,764 | 129 (2.7) | 60 (51 to 71) | Reference |
| HIV Positive |  |  |  |  |
| Clinical diagnosis | 2,228 | 303 (14) | 330 (295 to 369) | 1.35 (1.10 to 1.65) |
| Bacteriological diagnosis | 1,563 | 152 (9.7) | 229 (196 to 269) | Reference |
| **Age group** | | | | |
| 18 to 30 years |  |  |  |  |
| Clinical diagnosis | 1,772 | 99 (5.6) | 129 (106 to 157) | 2.10 (1.51 to 2.92) |
| Bacteriological diagnosis | 2,460 | 60 (2.4) | 55 (43 to 71) | Reference |
| 31 to 40 years |  |  |  |  |
| Clinical diagnosis | 1,676 | 123 (7.3) | 170 (143 t0 203) | 1.78 (1.34 to 2.37) |
| Bacteriological diagnosis | 1,858 | 74 (4.0) | 90 (72 to 113) | Reference |
| 41 to 50 years |  |  |  |  |
| Clinical diagnosis | 1,153 | 132 (11) | 274 (131 to 325) | 2.07 (1.51 to 2.84) |
| Bacteriological diagnosis | 1,001 | 55 (5.5) | 123 (94 to 160) | Reference |
| 51+ years |  |  |  |  |
| Clinical diagnosis | 1,871 | 285 (15) | 375 (334 to 421) | 1.71 (1.34 to 2.19) |
| Bacteriological diagnosis | 1,065 | 96 (9.0) | 206 (169 to 252) | Reference |
| PY-Person years, *the analysis excludes 140 patients with unknown HIV status | | | | |

Supplementary Table 9: **TB treatment outcomes after six months of anti-TB treatment stratified by type of TB.**

| **TB treatment outcome** | Pulmonary TB | | Extrapulmonary TB | |
| --- | --- | --- | --- | --- |
|  | **Clinical (N=5,009)** | **Bacteriological (N=6,330)** | **Clinical (N=1,463)** | **Bacteriological (N=54)** |
| Treatment success | 4,088 (82) | 5,285 (83) | 1.188 (81) | 40 (74) |
| Treatment failure | 5 (0.10) | 93 (1.5) | 0 | 0 |
| Died | 495 (9.9) | 276 (4.4) | 144 (9.8) | 9 (17) |
| Defaulted/Lost-to-follow-up | 295 (5.9) | 469 (7.4) | 94 (6.4) | 2 (3.7) |
| Transfer out | 126 (2.5) | 207 (3.3) | 37 (2.5) | 3 (5.6) |
| *Proportion of treatment success defined following WHO guideline, #Proportion of treatment success defined following WHO guideline. | | | | |

Supplementary Table 10: **Multivariate analysis of TB treatment outcomes associated with diagnosis of TB at time of starting anti-TB treatment stratified by TB type.**

| **TB treatment outcome** | Pulmonary TB | | Extrapulmonary TB | |
| --- | --- | --- | --- | --- |
|  | **Adjusted SHR**  **(95% CI)** | **P-value** | **Adjusted SHR (95% CI)#** | **P-value** |
| Treatment success | 1.02 (0.81 to 1.80) | 0.36 | 1.46 (0.42 to 5.07)^++^ | 0.55 |
|  | **Crude HR**  **(95% CI)** |  | **Adjusted HR (95% CI)#** |  |
| Treatment failure$ |  |  |  |  |
| Died | 7.06 (2.79 to 12.9) | <0.001 | 0.16 (0.01 to 3.09)^+++^ | 0.23 |
| Defaulted/Lost-to-follow-up^+^ | 0.98 (0.84 to 1.15) | 0.84 | 2.57 (0.59 to 11.2) | 0.21 |
| Transfer out | 0.89 (0.70 to 1.13) | 0.34 | 0.77 (0.19 to 3.07) | 0.71 |
| #Adjusted for *a priori* confounders: age, gender, patient type, TB type (P/EP), HIV status, treatment regimen and BMI groups, SHR; sub-distribution hazard ratios from Fine & Gray competing risk regression model, HR; Hazard ratios from Cox proportion regression models, +Hazard ratios are from the Gompertz parametric regression model, $No measures of association was estimated for treatment failure because of obvious bias in classifying the clinically diagnosed patients who had a negative sputum test when starting treatment, ++the adjusted regression models included the HIV interaction term, +++the adjusted regression models included the HIV, age interaction terms. | | | | |
